# A Singular Case of a Large Encysted Ossification Arising within the Spleen, and Attached to the Left Lobe of the Liver, Accompanied with Hydrothorax and Other Morbid Appearances

**Published:** 1821-01

**Authors:** R. W. Bampfield

**Affiliations:** Surgeon.


### *A singular Case of a large Encysted Ossification arising within the Spleen, and attached to the Left Lobe of the Liver, accompanied with Hydrothorax and other Morbid Appearances.* By R. W. Bampfield, Esq. Surgeon.

PATRICK M'CORMICK, ætatis 53, was received into the parish infirmary of Covent Garden, on the 31st of August, 1816. In giving a history of his complaints, he stated that he had been a porter and corker to wine-cellars for several years, and had been frequently tipsy. During seven or eight years he has been sick in the morning after eating, and frequently vomited: during the last two years he almost invariably experienced sickness, and pain about the large extremity of the stomach, after eating or drinking, which terminated in vomiting. The appetite was lost, the stomach flatulent, the ingesta turned sour on his stomach, and he felt an unusually uneasy sensation in it, which he firmly believed to originate from the presence of some cork, which he thought he had swallowed in his occupation of corking bottles. His bowels have been generally costive. He has five times fallen down in "fits," while carrying weights in the street. A short and difficult respiration has gradually increased, more especially for the last three years, on going up any ascent, or after any great exertion. For five years he has been affected with a winter cough and pain of the left side. He has been a stout athletic man, but has been seven or eight years gradually declining in strength and flesh, and subject to œdema of the feet.

On the 31st of August, he complained of severe orthopnœa, and inability of making a full inspiration; of a constant fixed pain of the left side, which greatly impeded respiration, and was referred to the false ribs. He could lay down on the affected side, or on the back with the head and shoulders raised; but he felt uneasiness like suffocation, and an anxious respiration, when he turned on his right side. He was affected with a cough, alternately dry and moist, but hard. The pulse was unusually hard and strong, beating about eighty-four strokes in a minute. His bowels were obstinately constipated; pyrexia was present; vomiting was frequent; he had anorexia; his cheeks were of a slight purple hue, and his expression of countenance denoted anxiety and suffering. The quantity of urine did not deviate from the healthy, but more than one-half of it coagulated by heat alone.

Mittatur sanguis ad remissionem doloris. Saline purgatives were prescribed, but every dose was rejected from the stomach, which rendered it necessary to commute the fluid for the solid form of purgative, and calomel and jalapæ pulvis were administered in doses repeated at proper intervals; but they did not operate copiously until two days had elapsed. The bleeding and purging procured considerable relief from pain. The blood drawn exhibited a thick coriaceous buff, and was cupped. A large blister was applied to the left side; and saline medicines, with acetum scillæ et ipecacuanha, were given. Low diet was ordered.

The hydrargyri submurias, taken as a purgative to the quantity of ten grains only in two days, excited a free salivation; which was not intended, but had the happy effect of enabling the bowels to act subsequently without any assistance from purgatives.

It will, perhaps, be only necessary to state generally, that the breathing and pain were in some degree relieved by the medicines above mentioned, and a succession of blisters to the left side. The pulse continued hard and strong, varying from 72 to 84. The orthopnœa and incapability to make a full inspiration continued. Latterly, he could only lay on his back with composure, with his head and shoulders more and more raised, until they reached the vertical position; and, finally, he could only breathe with his body inclined forwards. He every day vomited nearly the whole of what he had swallowed, more especially if taken in considerable quantity. At his own request the bleeding was thrice repeated, and afforded a transitory relief: the blood always exhibited the same inflammatory appearance.

On the 20th of September, the Tr. digitalis was prescribed instead of the squills; but it occasioned such a constant sickness and vomiting as induced me to omit it, and resume the use of squills: he, however, thought himself so much better after the use of digitalis as to sit up. To relieve the stomach affection, bitters with alkali were given for a day, at two distinct periods, but they instantly aggravated all the symptoms of hydrothorax; and, when the extract of gentian with soda exsiccata was given, their effect was so injurious as to induce the patient to suspect some very narcotic drug had been administered, and he desisted from their use after taking ten grains of the extract and five of the soda.

On the last days of September, and until he died, the pain of the left side, (for he never complained of any in the right,) the orthopnœa, frequent vomiting, inability of lying down, and the œdema of the lower extremities, increased. On the 1st of October, he could only breathe with difficulty by inclining himself forward in bed; his respiration was quick and anxious, and his pulse about 106. On the 2d and 3d, he could only perform respiration by resting his arms extended on the back of a chair, or on a pillow laid on the table, and inclining his body forward: his lower extremities swelled hourly, became quite anasarcous, and of an enormous size before death.

The body was opened by Mr. Coulthred, surgeon, Borough-road, and myself, on the 4th of October, the day after he died. The thorax was first examined. The pericardium contained about ℥iiſs of a serous fluid. The heart was larger than usual, and contained six coagula of blood, formerly termed polypi. Its fibres were flaccid, and of an amber colour. The coats of the arteries arising from it had a similar appearance, were soft, and had lost their usual elasticity. In the left cavity of the thorax there were four pints of a serous fluid. To the pleura costalis were adhering several pieces of coagulable lymph, transparent and of a gelatinous consistence, and some loose portions floated on the surface of the serum. The left lung was of a purple colour, not more than one-third of its natural size, and was attached to the anterior and posterior parts of the pleura costalis by membranous bands and close adhesions. The right cavity of the thorax contained two pints of fluid similar to the left, and similar portions of coagulable lymph. The right lung was larger than the left, but adhered by similar means to the whole length of the mediastinum. It was also of a dark-purple hue.

The cavity of the abdomen contained some serous fluid, in which were floating pieces of coagulable lymph, similar to what were met with in the lungs. The spleen exhibited a very singular morbid appearance; the other viscera were healthy. From the centre of the substance of the right end of the spleen arose a spherical bony tumor, resembling in size and figure the head of a fetus of seven months old, which at its right hemisphere was attached to the left lobe of the liver, which it had stretched and elongated. The long diameter of the tumor is three and a half inches, the short three inches; its largest circumference about eleven inches. The average thickness of the bone is one line, and it is covered, in many parts, with cartilage of various degrees of thickness. The tumor is hollow, and contained seven ounces of a fluid, chiefly serum, in which were floating small particles of a cetaceous substance, shining like mica. Its external covering, or periosteum, appears to be an elongation of the peritoneal covering of the spleen, where it is not enveloped by the substance of that viscus; and its internal lining appears to be a secreting membrane proper to itself. One-half of its external surface is covered by the substance of the spleen; the other by peritoneal membrane, and, generally speaking, by cartilage; the left lobe of the liver was united to the latter portion of the tumor, or right hemisphere, by adhesion of its peritoneal membrane, and by membranous bands.

The tumor adhered to the peritoneum behind, by which it was fixed in its position posterior to the stomach.

The right portion of the spleen is irregularly distributed over the section of the tumor imbedded in it, and gradually diminishes in thickness, until its tenuity is so small as to be lost in the membrane covering the right section of it. The spleen would probably be of its natural size if separated from the bone. The right end of it, and the portion covering the bone, were of a natural colour; the left end was of a dull flesh colour. The preparation is in Mr. Brooks' museum. From my own researches, and the observations of some learned physicians and surgeons who have seen this morbid appearance of the spleen, it is inferred that there is not a similar one on record.

The laws of nature, in the organization and growth of morbid as of natural parts, are equally concealed from human wisdom, and, perhaps, can only be known to nature's God; yet, from the little knowledge we possess on this subject, we may venture to offer a speculative opinion that this tumor was, probably, in its origin, a membranous cyst formed in the right portion of the spleen, the layers of which were gradually converted into cartilage and bone in its progressive growth, during which it irregularly separated the substance of the spleen, spread it on its surface, and, on reaching its peritoneal covering, extended it, and appropriated it to itself as a periosteum. That this tumor originated in the substance of the spleen, would seem evident from two circumstances: first, that the section of the tumor imbedded in the spleen is closely connected to its substance, and has no membranous covering; secondly, because the peritoneal coat of the spleen is extended over the section of the tumor exterior to the substance of it, which is also irregularly divided over its circumference.

The immediate cause of death appears to have been an accumulation of water in the chest and pericardium; but, how far the morbid structure of the spleen might have operated as a remote cause of hydrothorax, I cannot offer any reasonable conjecture. The severe affections of the stomach may be attributed to the mechanical pressure of this rude mass, more particularly when the stomach was distended, and is another proof that very untractable affections of the stomach may be caused by morbid derangement of the viscera contiguous to it. The coagulable urine observed in this case proves that it may be present in dropsy, when the viscera are both diseased and deranged.

*37, Bedford-street, Covent Garden;*
*October 8th, 1820.*

